# Surface and adsorption characteristics of three elastin-like polypeptide coatings with varying sequence lengths

**DOI:** 10.1007/s10856-012-4772-6

**Published:** 2012-09-30

**Authors:** Elizabeth M. Srokowski, Kimberly A. Woodhouse

**Affiliations:** 1 Department of Chemical Engineering and Applied Science, University of Toronto, Toronto, ON Canada; 2 Institute of Biomaterials and Biomedical Engineering, University of Toronto, Toronto, ON Canada; 3 Department of Chemical Engineering, Queen’s University, Kingston, ON Canada

## Abstract

The surface properties of a family of elastin-like polypeptides (ELPs), differing in molecular weight and sequence length, were investigated to understand how the nature of the polypeptide film might contribute to their thrombogenic profile. Physical adsorption of the ELPs onto Mylar increased surface wettability as the sequence length decreased while X-ray spectroscopy analysis showed an increasing amide content with sequence length. Chemical force microscopy analysis revealed that the ELP-coated surfaces displayed purely hydrophilic adhesion forces that increased as the ELP sequence length decreased. Adsorption isotherms performed using the quartz crystal microbalance with dissipation, showed that the surface coverage increased with ELP sequence length. The longer polypeptides (ELP2 and ELP4) also displayed higher specific dissipation values indicating that they established films with greater structural flexibility and associated water content than the shorter polypeptide, ELP1. Additionally, the stability of the ELP coating was lower with the shorter polypeptides. This study highlights the different surface properties of the ELP coatings as well as the dynamic nature of the ELP adsorbed layer wherein the conformational state may be an important factor contributing to their blood response.

## Introduction

Despite tremendous research on materials for blood contacting devices such as stents, vascular grafts and ventricular assist devices, the development of a truly blood compatible material still remains unsolved [1, 2]. A major clinical concern for blood contacting biomaterials is surface-induced thrombus generation [3–5]. The rapid adsorption of various plasma proteins at the blood-material interface is believed to be the initial factor influencing thrombus generation. In fact, the adsorbed protein layer may trigger a complex series of events including platelet activation and adhesion, as well as coagulation and complement activation, leading to thrombus generation [4, 6, 7]. These events may also be further complicated by the initiation of inflammation and wound healing responses, ultimately affecting the biocompatibility of the biomaterial [8].

To improve the hemocompatibility of blood-contacting biomaterials, several strategies have focused on manipulating the surface properties of the biomaterial. In broad terms, these approaches may involve surface endothelialization, surface passivation or bioactive coatings [9, 10]. Recently, there is growing evidence in the literature demonstrating the potential the elastin protein, a constituent structural protein of the vascular wall, has to enhance the hemocompatibility of biomaterials [11, 12]. Studies have revealed that surface coatings with the elastin protein and elastin-derived molecules are capable of inhibiting thrombosis related complications in vivo [13, 14] as well as enhancing endothelial cell interaction in vitro [15, 16]. Studies from our own laboratory [14, 17] have also demonstrated that when our family of recombinant elastin-like polypeptides (ELPs) is physically adsorbed onto polymeric substrates, the blood compatibility of a surface is improved. Our ELPs are based on the structure of the native elastin protein, tropoelastin that contains an alternating hydrophobic and cross-linking domain structure. Each ELP consists of exons 20, 21, 23 and 24 of the human aortic tropoelastin gene, but varies by molecular weight as well as by the number of alternating domain structures. Recently, we have shown through an in vitro study [17], that our family of ELP coatings can alter the magnitude of fibrinogen adsorption and platelet adhesion in an ELP sequence length dependent manner. Despite the compelling thromboresistant properties of the elastin protein and elastin-derived molecules, it remains unclear in the literature as to the factor(s) responsible for their low thrombogenicity. Moreover, it is currently unclear how the sequence length of our family of ELPs influences the nature of the adsorbed polypeptide film, which ultimately affects the interactions occurring at the blood-material interface. Thus, the overall aim of the present study was to characterize the polypeptide-surface interface of a family of ELPs physically adsorbed onto the surface of Mylar, in order to identify potential factor(s) influencing their surface bioactivity. A combination of techniques was utilized to characterize the surface properties of three ELP coatings (ELP1, ELP2 and ELP4) including goniometry, X-ray spectroscopy (XPS) and atomic force microscopy (AFM). Adsorption isotherms were also performed using the quartz crystal microbalance with energy dissipation (QCM-D) to obtain information in real-time and in situ regarding the polypeptide’s adsorption process.

## Materials and methods

### Materials

All chemical reagents were purchased from Sigma-Aldrich Canada (Oakville, ON, Canada), unless otherwise noted. Mylar (300 Å) sheets were obtained from Active Industries (Clifton Park NY). Mylar is a noncrystalline form of polyethylene terephthalate (PET). Prior to use, all Mylar surfaces were pre-treated (pMylar) with several methanol rinses and then equilibrated in plain Tyrode’s buffer (0.14 M NaCl, 2.6 mM KCl, 11.9 mM NaHCO_3_, 0.4 mM NaH_2_PO_4_, pH 7.4), overnight at room temperature to ensure a clean initial surface, unless otherwise noted. All buffers were made with deionized water with resistivity 18 MΩ cm.

### Elastin-like polypeptides

**Fig. 1:**
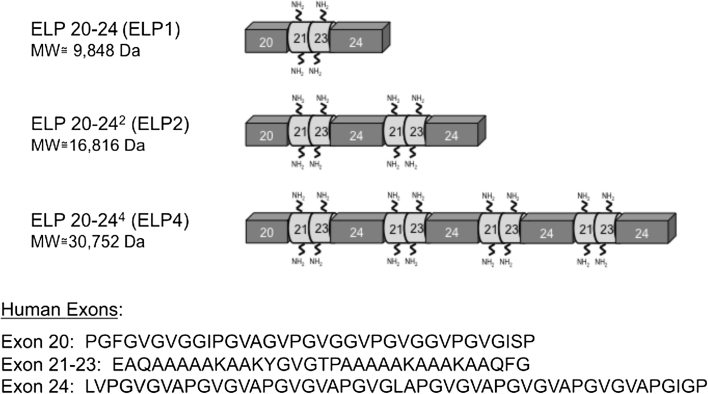
Schematic representation of the ELPs sequences used in the current adsorption study along with their molecular weights. The amino acid composition of each exon in the ELP sequences is also listed

Our family of recombinant ELPs contain the alternating domain structure of human aortic tropoelastin consisting of the hydrophobic domains 20 and 24 separated by cross-linking domains 21 and 23 in the form 20-(21-23-24)_*n*_, where *n* indicates the number of domain 21, 23 and 24 repeats [18, 19]. The three ELPs investigated in this study consist of repeating hydrophobic and cross-linking domains in a similar fashion to that of tropoelastin, but differ by molecular weight and number of repeating domains. Figure 1 shows a schematic representation of the polypeptide sequences used in this study. ELP20-24 [ELP1] contains two hydrophobic domains (exons 20 and 24) flanked by one cross-linking domain (exons 21 and 23), ELP20-24^2^ [ELP2] contains three hydrophobic domains (exons 20 and 24) flanked by two cross-linking domains (exons 21 and 23), and ELP20-24^4^ [ELP4] contains five hydrophobic domains flanked by four cross-linking domains [18, 19]. The expression, production and purification of the three ELPs is described elsewhere [17, 18]. Amino acid composition and molecular weight of each of the polypeptides was determined by amino acid analysis and MALDI Q-TOF mass spectrometry, respectively, using the Advanced Protein Technology Center (Hospital for Sick Children, Toronto, ON). All ELPs used in this study had a minimum purity of 90 %.

### Material characterization

#### ELP adsorption and desorption surface treatment

ELP coatings were achieved by physical adsorption onto Mylar, as previously described [17]. In particular, 1 mg/mL ELP bulk solutions (dissolved in Dulbecco’s phosphate buffer saline, PBS) were prepared 1 day prior to adsorption and stored at 4 °C until use in order to allow for sufficient mixing and equilibration of the polypeptide into solution. Taking into consideration the different molecular weights of the three ELPs, the bulk molarities of the ELP solutions were 102 nM ELP1 (9.8 kDa), 60 nM ELP2 (16.8 kDa) and 33 nM ELP4 (30.8 kDa). The following day, the ELPs (0.3 mg/cm^2^, or 30 nmol/cm^2^ ELP1, 18 nmol/cm^2^ ELP2 and 10 nmol/cm^2^ ELP4 in PBS buffer) were physically adsorbed onto pMylar for 3 h at room temperature (~22 °C). Following adsorption, the surfaces were gently rinsed three times with plain Tyrode’s buffer (pH 7.4) before being analyzed for protein adsorption, unless noted otherwise. At the following coating conditions, ELP self-aggregation or coacervation was not found, as confirmed previously through coacervation profile studies [17].

To assess the stability of the coatings, the adsorbed ELP surfaces (34 mm in diameter films) underwent a shear-based desorption treatment where the surfaces were exposed to similar shear flow conditions from a cone and plate device [3, 17] used for in vitro hemocompatibility tests. In particular, the ELP-coated surfaces were placed into the wells of the cone and plate viscometer along with 0.9 mL of PBS. The cones were lowered and allowed to rotate at 200 rpm (or 300/s) for 15 min at room temperature. Afterwards the surfaces were gently rinsed three times with plain Tyrode’s buffer (pH 7.4) before being analyzed.

#### Water contact angle analysis

The water–air contact angle measurements were performed to determine the relative hydrophobicity of the adsorbed and desorbed ELP-coated and uncoated Mylar surfaces. Measurements were taken on 34 mm diameter samples of Mylar, pMylar, and the adsorbed and desorbed ELP-coated Mylar surfaces (aELP and dELP, respectively). Prior to analysis, all surfaces were rinsed three times with deionized water at room temperature and then placed in a vacuum oven for drying overnight at room temperature (~22 °C). Immediately following the drying procedure, the advanced water contact angle, θ_adv_ was measured with a VCA Optima XE goniometer (AST Products Inc, Billerica, MA) using the sessile drop technique with deionized water, as previously described [17]. Three readings were performed at random locations on each sample surface. A minimum number of 15 drops was used for the control surfaces of Mylar and pMylar, while a minimum number of 25 drops was used for the adsorbed and desorbed ELP-coated surfaces.

#### Elemental and chemical surface analysis, XPS

XPS of all surfaces was conducted with 1 cm^2^ samples. Spectra were acquired on the Thermo Scientific K-Alpha XPS spectrometer using a monochromatic AlK_α_ X-ray source with a take off angle (toa) (relative to the surface) of 90° at the SI-Ontario facility, University of Toronto. The energy scale for all spectra was corrected to place the main C 1s feature (C–C) at 285 eV. Atomic ratios for the ELP-coated and uncoated surfaces were obtained from spectra collected at low-resolution, while the chemical composition was determined from high-resolution mode.

#### Surface topography and composition, AFM

AFM measurements on the ELP-coated and uncoated (pMylar) surfaces (14 mm in diameter) were acquired at ambient temperature in contact mode on a Digital Instruments Nanoscope IIIa Multimode SPM (Digital Instruments, Santa Barbara) equipped with an “E” scanner in situ. Ex situ measurements (in PBS buffer) were obtained using a contact/tapping mode fluid cell where the substrate was sealed with a Teflon O-ring and fitted with inlet and outlet tubing to allow exchange of solutions in the cell during assessment, as previously described [20]. Images and force curve measurements were obtained with chemically modified hydrophobic (–CH_3_) and hydrophilic (–COOH) silicon nitride AFM probes, with a nominal spring constant of ~0.58 N/m, used as supplied by the manufacturer (Novascan Technologies, Inc., #CT.AU.CH3 and #CT.AU.COOH, respectively). Deflections versus piezo extension curves were collected at three different frequencies of the approach/retract cycle (scan rate) (0.5, 1.0 and 5.0 Hz), with a scan size of 125 or 200 nm. A trigger threshold of 50 nm was applied to the ex situ cycles to limit the force applied on the probe to a maximum value of approximately 29 nN. A minimum of five measurements for each frequency was acquired for a set position on the substrate, and this was repeated for a minimum of three different positions on the substrate. In order to eliminate discrepancies between the samples, the same sample substrate was analyzed with the two different AFM probes. Similarly, to eliminate discrepancies between the AFM probes (i.e. differences in the radius of curvature for each probe) the same probe was used to analyze the four different substrates. This procedure was repeated at two separate trials. Images and force curves were analyzed with the Digital Instruments Nanoscope v5.30r3sr3 software (Veeco, Santa Barbara) and the force-curves processed with a custom-written Nanoscope Force Curve Analysis Software, SPMCON, by Dr. C.M. Yip, University of Toronto. From the generated force curves monitoring the deflection of the cantilever as a function of the piezo translation, the maximum force of adhesion (F_AD_), was computed based on the deflection during retraction of the tip from the sample using Hooke’s Law (i.e. F = −kx), where F is force, k is the nominal spring constant (0.58 N/m), and x is deflection. A minimum of 15 measurements at each frequency were analyzed to compute the mean and standard deviation of the F_AD_ for each sample surface.

#### QCM-D

**Fig. 2:**
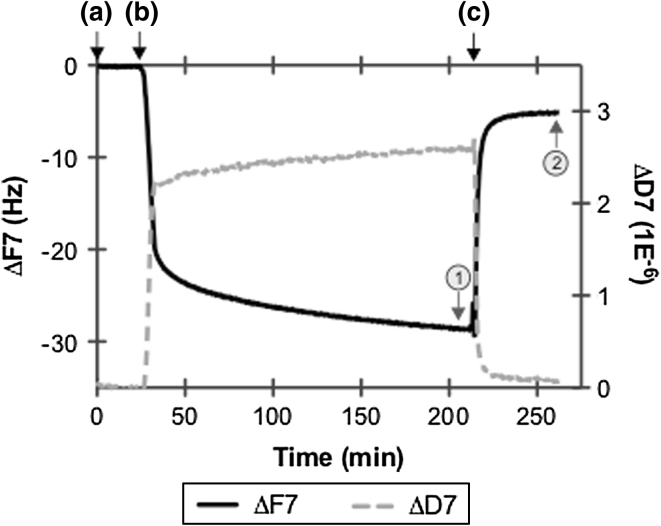
Representative real-time ELP1 adsorption isotherm (5.8 mg/mL, PBS at 22 °C) to the PET-coated QCM-D sensor, showing the change in frequency ΔF (7th overtone) and the change in dissipation ΔD (7th overtone) over the course of the experimental trial that included: *a* establishing a stable baseline in PBS at 50 μL/min for 5 min; *b* addition of ELP1 solution at 50 μL/min for 6 min followed by static adsorption for up to 3 h; *c* rinsing of surface with PBS at 50 μL/min for 30 min. *Points* *1* and *2* indicate where modeling for total and post-rinse ELP surface coverage was performed, respectively

ELP adsorption isotherms were carried out using a QCM-D that monitored the adsorbed polypeptide amount from a range of ELP bulk concentrations. QCM-D is a well-established technique for monitoring in situ and real-time adsorption and conformational changes of biomolecules at the surface. A change in mass due to the interaction of the biomolecule with the quartz crystal surface results in a change in the resonant frequency, ΔF. A change in the energy dissipation factor, ΔD, reflects a change in the viscoelastic properties of the adsorbed layer. In this study, PET-coated quartz crystals (5 MHz, 14 mm in diameter with an assumed active area of 78.5 mm^2^) were used, having ~50 μm PET thickness obtained from Q-Sense AB (Gothenburg, Sweden). ELP adsorption to the PET-coated quartz crystals was performed using the QCM-D Q-Sense E1 system at room temperature (22 °C) with a flow module attached to an Ismatec Reglo Digital pump for flow control. ELP adsorption from the various bulk concentrations—0.01, 0.1, 1.0, 5.8, and 11.8 mg/mL in PBS, was monitored at all of the crystal’s harmonic overtones by simultaneously recording the ΔF and the ΔD using the Q-Sense Q-Soft software. Each ELP bulk concentration was repeated at least three times. Prior to the start of an experiment, crystals were first equilibrated in the module in deionized water, followed by equilibration in PBS buffer for approximately 1 h. As shown in Fig. 2, a typical experiment consisted of the following steps: (a) establish a stable baseline in protein-free buffer (i.e. PBS) pumped at 50 μL/min; (b) introduce ELP solution to the surface pumped at 50 μL/min until a ΔF and corresponding ΔD was registered, at which point the ELP solution was allowed to pump through for an additional 6 min (this was the duration of time needed for the solution to fully exchange through the flow module) and then stopped to allow static adsorption for a maximum of 3 h; and finally (c) rinse surface with protein-free PBS at 50 μL/min for ~30 min. To measure total adsorption of the ELP, the mass per unit area (ng/cm^2^) (i.e. surface coverage) was modeled prior to rinsing with PBS buffer (see point 1 in Fig. 2). Alternately, the post-rinse ELP surface coverage was modeled at the end of the PBS buffer (see point 2 in Fig. 2). When the change in dissipation (ΔD) was below 5 % of the frequency shift (ΔF), suggesting the formation of a thin, rigid, uniform adsorbed film, the ELP surface coverage (total and post-rinse) was estimated using the Sauerbrey relation. This was primarily conducted for bulk concentrations lower than 1 mg/mL for most of the ELPs. Conversely, when the ΔD >5 % ΔF, suggesting the formation of a soft, viscous adsorbed film, the Sauerbrey equation underestimates the adsorbed mass [21]. In this case, the ELP surface coverage values were approximated using the Voigt-based model [22] with the combination of 5th–11th overtones within the Q-Tools data analysis software (Q-Sense, Gothenburg, Sweden). In the Voigt-based model [22], it is assumed that the crystal is covered by a uniform and homogenous viscoelastic film in contact with a semi-infinite Newtonian liquid under no-slip conditions. To model the QCM-D response using the Voigt-based model [22], the effective layer density was allowed to range from 1,050 to 1,250 kg/m^3^ for each ELP sequence and bulk concentration in order to account for the differences the molecular weights of the ELPs. In addition, the following parameter ranges were used to obtain the lowest χ^2^ value (indicating the best model fit): (i) layer viscosity 0.001–0.005 kg/m s; (ii) layer shear 10^3^–10^8^ Pa; and (iii) layer mass 10–5,000 ng/cm^2^. The reported average and standard deviation values for the estimated ELP surface coverage (ng/cm^2^) following total adsorption (i.e. after static adsorption for 3 h) and post-rinse adsorption were computed based on the outcome of either the Voigt-based model [22] or the Sauerbrey equation using the 7th overtone. Any additional analysis on the QCM-D response (i.e. ΔF and ΔD) was performed based on the 7th overtone, as it not only consistently exhibited minimum noise in the collected data, but whose values have been commonly reported [23–25].

To allow for crystal re-use, the crystal underwent a cleaning treatment immediately following the ELP adsorption run to remove the adsorbed ELP layer but to maintain the original PET coating. The cleaning treatment was adapted from Liu et al. [26], but in this situation used a surfactant-based cleaning agent, Deconex^®^ II Universal (TECHNOTRADE International, Inc. Manchester, NH), which has been previously used by other investigators to clean QCM sensors [27]. In particular, the crystal surface was exposed to a 3 % Deconex^®^ II Universal solution, at 50 μL/min for ~30 min at 37 °C, followed by rinsing with deionized water at 50 μL/min for ~30 min at 37 °C while monitoring ΔF and ΔD. The crystal was then taken out of the QCM-D module, rinsed repeatedly with deionized water and stored at least overnight at room temperature in deionized water. Upon being re-used, the crystal was rinsed with 99 % ethanol, followed by deionized water before being dried with argon gas. The crystal was mounted into the QCM-D module and rinsed with deionized water for a minimum of 60 min at 50 μL/min at 22 °C. A deionized water baseline was then obtained for the crystal, and stitched to the original deionized water baseline for that particular crystal. Using the Q-Tool data analysis software, the thickness (nm) of the crystal surface layer was monitored using the Sauerbrey equation for the 7th overtone since ΔD >5 % ΔF. The thickness of the cleaned crystal surface was compared to the original reading to ensure no deviation; otherwise the cleaning treatment was repeated. In total, a maximum of five cleaning treatments were allowed for each crystal before it was no longer used.

### Statistical analysis

All measurements were performed in triplicate on each type of sample unless otherwise specified. One-way analysis of variance (ANOVA) followed by a Bonferroni post hoc multiple-comparison test was used for comparison using GraphPad Prism 5 statistical software. For comparison between two data groups, an unpaired two-tail Student *t* test was used. In all tests, *P* values of less than 0.05 were considered statistically significant. All quantitative data is represented as mean ± standard deviation (SD).

## Results and discussion

### Wettability

**Fig. 3:**
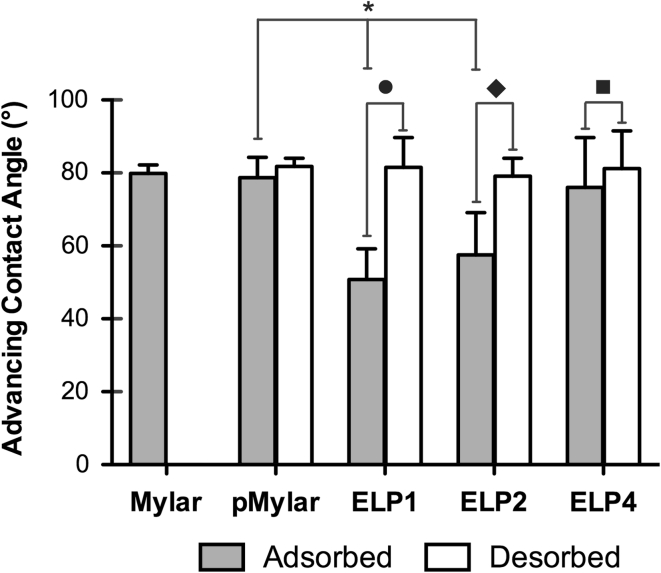
Summary of the wettability [characterized by the water advancing angle (°)] for the adsorbed ELP-coated and uncoated Mylar surfaces (Mylar and pMylar) as well as following a desorption treatment. *Asterisk* indicates a significant change in wettability in comparison to pMylar (*P* < 0.05). Following adsorption, the wettability for each of the ELP-coated surfaces was significantly different from one another (*P* < 0.05). *Filled circle*, *filled diamond*, and *filled square* indicate a significant change in wettability following the desorption treatment in comparison to the adsorbed surface for ELP1, ELP2 and ELP4, respectively

The advancing water contact angle θ_adv_, characterizing the wettability of the ELP-coated surfaces after the adsorption as well as the desorption treatment, along with the control surfaces of Mylar and pMylar, is summarized in Fig. 3. Both Mylar and pMylar (post methanol rinses and incubation in plain Tyrode’s buffer) were found to be similar in hydrophilicity having an θ_adv_ = ~80°, a value commonly reported in the literature for Mylar/PET [28]. Physical adsorption of the family of ELPs onto pMylar increased the wetting properties of the surface significantly with the shorter polypeptides—ELP1 (50.8 ± 8.4°) and ELP2 (57.5 ± 11.7°) but not with the longest polypeptide, ELP4 (76.2 ± 13.7°); a trend consistent with our previous study using a different goniometer [17]. The increased wettability of the surface is also in agreement with values reported by others in the literature for substrates modified with other elastin-based molecules [13, 29]. Moreover, the wettability for each of the ELP-coated surfaces was statistically different from one another and inversely coincided with the polypeptide sequence length: ELP1 > ELP2 > ELP4.

Following the desorption treatment, a significant decrease in wettability for each of the ELP-coated surfaces was found, with values ranging in the same vicinity as for the uncoated control of pMylar, as shown in Fig. 3. The greatest magnitude of change in wettability [i.e. difference in θ_adv_ between the desorbed and adsorbed (dELP − aELP) surface] among the ELP surfaces ranged from ELP1 (~31°) > ELP2 (~21°) > ELP4 (~6°), suggesting that the stability of the coating with the shorter polypeptides is less than with the longer polypeptide sequences.

### Surface chemistry

The elemental and chemical composition of the adsorbed and desorbed ELP-coated surfaces were evaluated by XPS at the take off angle (toa) (relative to the surface) of 90°, as summarized in Table 1. From the high-resolution spectra, the C1s envelope was fit to four peaks corresponding to C1=C–C/C–H (~285 eV), C2=C–O/C–N (~286 eV), C3=N–C=O (~287 eV), and C4=O–C=O (~289 eV), in accordance with literature values [30, 31]. The atomic composition of the uncoated Mylar surfaces (i.e. pMylar and Mylar) was similar to the theoretical value (i.e. C:N:O ratio of 2.5:0:1). However, traces of nitrogen content (N1s) were found possibly due to additives added during commercial processing of the Mylar film. Adsorption of the family of ELPs (aELP) resulted in a visible increase in both nitrogen content [N(1s)] and amide content (C3) relative to the uncoated Mylar control, confirming the modification of the surface with the ELPs. Among the ELP-coated surfaces, ELP1 generally displayed lower levels of nitrogen and amide content compared to the longer ELP-coated surfaces. This may suggest that less of ELP1 was present on the Mylar surface than with the other ELPs. The ratio of ester (C4) to amide (C3) was also found to vary among the ELP-coated surfaces, with ELP1 exhibiting a higher ratio compared to the other ELPs. This finding may suggest that the distribution and/or conformation of the adsorbed ELPs may not be the same on the surface, in particular between ELP1 and the longer ELPs. Furthermore, it was interesting to find that the most wettable ELP1-coated surface generally exhibited lower levels of amide content then the other ELP-coated surfaces, as surfaces having more amides are typically more hydrophilic than those with more esters [32]. Nonetheless, it is important to acknowledge that the overall structure of elastin has been suggested to be highly labile, with intrinsic flexibility to its backbone [33, 34] and a structure that is dynamically interchanging according to different microenvironments [35, 36]. In particular, a recent study by Le Brun and colleagues [35] demonstrated that the protein structure of surface-bound tropoelastin in air is altered, resulting in a collapsed structure from that in an aqueous environment. Consequently, under an aqueous environment, the detected differences in wettability may in part be attributed to variations in the ELP conformation among the ELP-coated surfaces that would not be reflected in the dry state of the XPS measurements.

**Table 1:** XPS summary of the elemental (low-resolution) and chemical (high-resolution) composition (%) at the take off angle of 90° for the ELP-coated and uncoated surfaces following physical adsorption (denoted by ‘a) and the desorption treatment (denoted by ‘d’)

Surface (*n* > 3)	N (1s)	O (1s)	C (1s)					
	Total	Total	Total	C–H/C–C ‘C1’ (~285 eV)	C–O/C–N ‘C2’ (~286 eV)	N–C=O ‘C3’ (~287 eV)	O–C=O ‘C4’ (~289 eV)	Ratio ester/amide
Mylar	0.4 ± 0.3	27.1 ± 1.0	71.8 ± 0.8	59.9 ± 6.0	18.6 ± 1.4		15.8 ± 1.6	
pMylar	2.1 ± 1.8	26.0 ± 0.5	70.4 ± 0.4	58.4 ± 2.6	17.3 ± 2.3		14.6 ± 0.9	
aELP1	4.5 ± 0.7	23.9 ± 1.1	70.6 ± 1.7	52.3 ± 3.2	17.6 ± 1.1	5.6 ± 1.1	12.9 ± 0.9	2.3
aELP2	7.6 ± 1.2	21.9 ± 1.6	69.8 ± 0.9	49.3 ± 5.0	16.6 ± 2.1	8.7 ± 2.0	10.8 ± 1.3	1.2
aELP4	7.1 ± 1.0	22.1 ± 1.0	70.5 ± 0.8	50.5 ± 2.7	18.7 ± 2.1	7.4 ± 1.3	11.9 ± 1.1	1.6
dELP1	3.2 ± 3.9	19.3 ± 2.3	75.8 ± 6.3	58.7 ± 7.2	12.1 ± 3.5	2.7 ± 0.9	8.4 ± 1.7	3.1
dELP2	4.4 ± 1.2	21.3 ± 3.2	73.6 ± 4.6	57.5 ± 2.1	14.0 ± 2.8	5.8 ± 1.4	10.0 ± 1.5	1.7
dELP4	4.6 ± 2.5	19.3 ± 2.9	74.5 ± 4.8	57.7 ± 3.3	13.3 ± 1.8	6.3 ± 1.9	7.9 ± 1.9	1.3

Values represent the means of the number of samples analyzed per surface (*n* > 3) ±SD

Following the shear-based desorption treatment, a decrease in the nitrogen content was observed for each of the ELP coatings (dELPs) in comparison to their adsorbed coating counterpart (aELPs). Additionally, a decrease in both the C3 and C2 signal was observed, the latter being partially attributed to a possible decrease in amine content (C–N) on the surface. Surprisingly, following the desorption treatment a decrease in the C4 (ester) signal occurred. Although it is uncertain, this may be due to either a patchy coating being left behind on the surface, and/or the amino acids rich in amine and ester content of the ELP sequence (residing mostly in the cross-linking domains) possibly being further buried away from the outermost surface. Nonetheless, the amide content (C3) was used to confirm the presence of the ELP following the desorption treatment, since a nitrogen signal was detected in the Mylar surfaces. Interestingly, the amide content was higher for the longer ELPs indicating that more of the ELP2 and ELP4 coating was retained on the surface than with ELP1. In fact, the magnitude of change in amide content following the desorption treatment suggested that ~52 % of ELP1 desorbed from the surface, followed by ~33 % of ELP2 and ~15 % of ELP4, a coating retention trend that coincides with the wettability analysis. Additionally, the magnitude of displaced ELP appears to be in the similar vicinity to that of previous study performed in our laboratory [14] with ELP2-coated surfaces, where ~40 % of the polypeptide desorbed from the surface following overnight incubation in buffer under static conditions.

### Surface topography and adhesion force

**Fig. 4:**
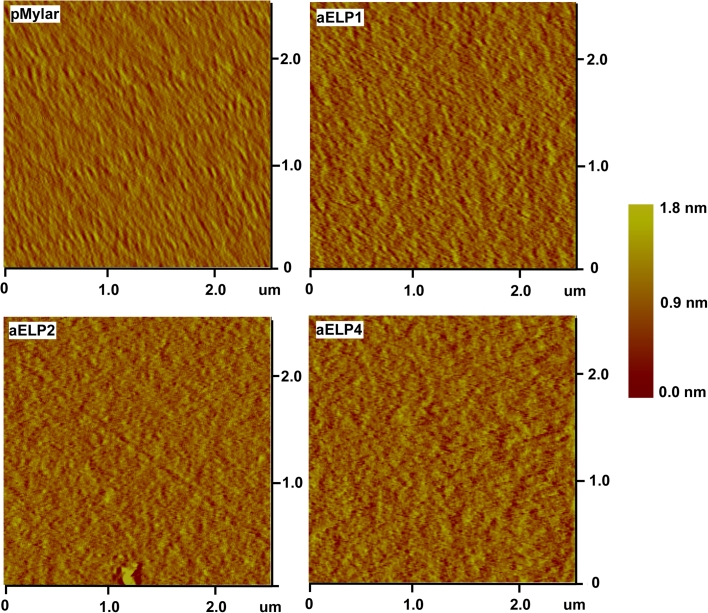
Representative ex situ (in PBS) AFM 2D topographic images (2.5 × 2.5 μm, *z* = 1.8 nm) for the ELP-coated and uncoated pMylar surfaces

Surface topography of the ELP-coated surfaces ex situ (in PBS buffer) was assessed with AFM in contact mode, as displayed in Fig. 4 showing representative topographical 2D images of the surface samples. In general, there was no discernable difference in surface features between the uncoated (pMylar) and the ELP-coated surfaces, with each of the ELPs appearing to adsorb to the underlying substrate producing a conformal coating.

**Fig. 5:**
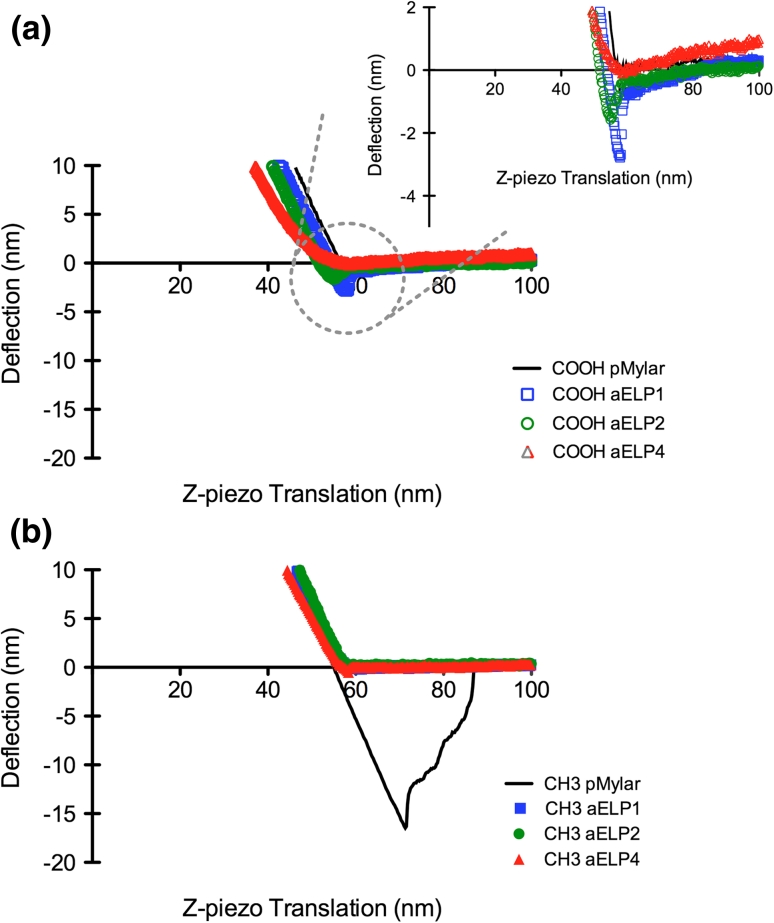
Typical retract traces for the ex situ (in PBS buffer) force-curve measurements at a scan rate of 0.5 Hz for the ELP-coated and uncoated surface, pMylar, using **a** the hydrophilic (–COOH) or **b** the hydrophobic (–CH_3_) tip. *Inset* on the force-curve plot for the hydrophilic tip **a** is a zoomed in view of the *outlined circle*

**Fig. 6:**
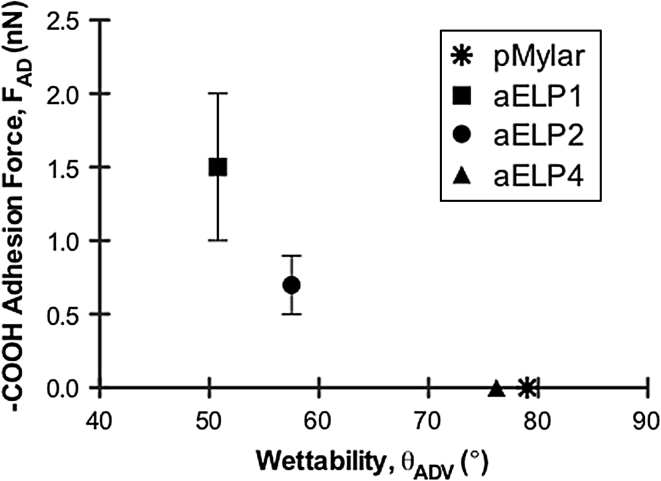
Comparison of the wettability of the ELP-coated surfaces as a function of the hydrophilic adhesion force, F_AD_. Data represented as the mean ± SD, *n* ≥ 15

Ex situ force-curve measurements using chemically modified AFM-tips of either hydrophobic (–CH_3_) or hydrophilic (–COOH) functionality were used to gain a better understanding of the chemical composition of the ELP coatings as well as to monitor the adhesive strength of the ELPs following adsorption to pMylar. Representative force-curves in raw data form for the retract phase of the curve (approach phase was similar for all of the conditions and therefore not shown) is illustrated in Fig. 5, using the hydrophobic and hydrophilic tips. Unlike the retraction traces using the hydrophobic tip that showed significant interaction only with the uncoated pMylar surface, the ELP-coated surfaces displayed some modest interaction with the hydrophilic tip. As summarized in Table 2, the greatest adhesion force (F_AD_) with the hydrophilic tip was obtained with the ELP1-coated surfaces (~1.2–1.7 nN), followed by the ELP2-coated surfaces (~0.6–0.7 nN), while the ELP4-coated surfaces showed no detectable hydrophilic adhesive force, similar to the uncoated pMylar surfaces. From our results, the trend in hydrophilic adhesion force is similar to the wettability trend where the shorter polypeptides established more wettable surfaces, as shown in Fig. 6. This suggests that a certain degree of molecular rearrangement of the labile polypeptide structure upon adsorption may indeed be occurring in the aqueous environment. Moreover, as elastin is commonly defined as a highly hydrophobic protein (hydropathy index increasing with ELP sequence length, ranging from 0.88 to 0.91 [17]), it was unexpected that there was no detectable interaction with the hydrophobic tip. However, it is plausible that the hydration layer or the hydrogen-bonded clathrate water structure known to surround the ELP molecules [24, 37] (playing a major role in the ELPs’ intrinsic capacity for self-aggregation or coacervation), may have shielded the surface from the hydrophobic tip, completely minimizing its interaction. Lastly, the narrow range of F_AD_ for the ELP-coated surfaces recorded at different spots on the samples at three different scan rates, suggests that the polypeptide coverage under the performed coating conditions is continuous, as illustrated by the AFM images.

**Table 2:** Summary of the approximate adhesion forces (F_AD_, nN) evaluated for the ELP-coated and uncoated surface of pMylar at varying scan rates using the hydrophilic (–COOH) and hydrophobic (–CH_3_) tips in PBS

Scan rate	0.5 Hz	1 Hz	5 Hz
	Adhesion force (nN)	Adhesion force (nN)	Adhesion force (nN)
–COOH			
pMylar	0.0 ± 0.0	0.0 ± 0.0	0.0 ± 0.0
aELP1	1.2 ± 0.5	1.6 ± 0.5	1.7 ± 0.4
aELP2	0.7 ± 0.2	0.7 ± 0.3	0.6 ± 0.1
aELP4	0.0 ± 0.0	0.0 ± 0.0	0.0 ± 0.0
–CH_3_			
pMylar	9.2 ± 4.9	16.7 ± 6.1	12.0 ± 5.5
aELP1	0.0 ± 0.0	0.0 ± 0.0	0.0 ± 0.0
aELP2	0.0 ± 0.0	0.0 ± 0.0	0.0 ± 0.0
aELP4	0.0 ± 0.0	0.0 ± 0.0	0.0 ± 0.0

Values represent the means of the number of samples analyzed per surface (*n* ≥ 15) ±SD

### Adsorption isotherms, conformation and hydration analysis

**Fig. 7:**
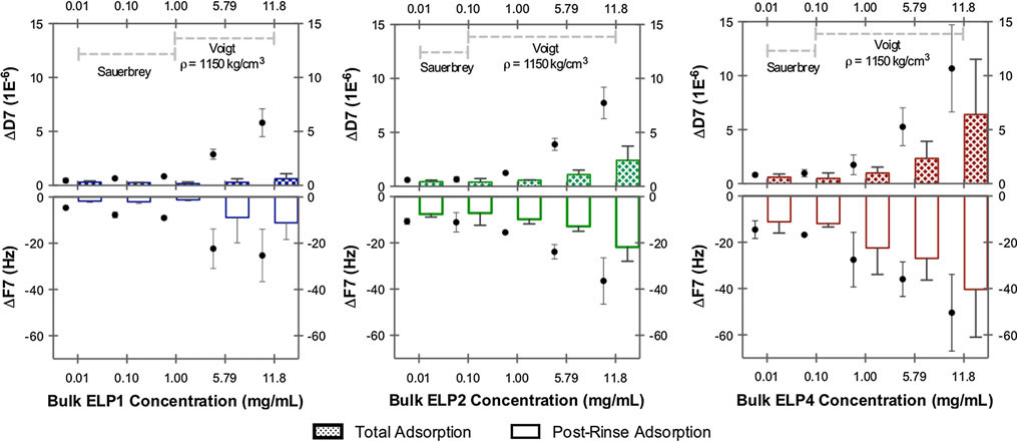
Summary of the ELP adsorption isotherms QCM-D response showing the changes in frequency (ΔF, Hz) and changes in dissipation (ΔD, 1E^−6^) following total adsorption (*circle*) after 3 h of static adsorption and following post-rinse adsorption (*bar*) after ~30 min of rinsing in PBS buffer. *Plots* also indicate when the Sauerbrey or the Voigt-based model [22] was used, as well as the optimized effective density (1,150 kg/cm^3^) used to estimate the ELP adsorbed mass (i.e. surface coverage) in Fig. 8. *Data* represented as the mean ± SD, *n* ≥ 3

Using the QCM-D, adsorption isotherms were carried out for each of the ELPs with a range of bulk concentrations from 0.01 to 11.8 mg/mL. The mid ELP bulk concentration of 5.8 mg/mL was equivalent to the bulk ELP surface density of 0.3 mg/cm^2^, used previously to establish the ELP coatings for in vitro hemocompatibility assessment [17]. Therefore, the adsorption properties at this ELP bulk concentration (5.8 mg/mL) will be highlighted. In general, as shown in Fig. 7, as the ELP bulk concentration increased, the change in frequency (ΔF) and change in dissipation (ΔD) (7th overtone) correspondingly increased. Furthermore, the magnitude in the ΔF and the ΔD increased with the ELP sequence length (and therefore molecular weight). Adsorption of ELP4 to the PET-coated sensor resulted in the greatest ΔF and ΔD in comparison to the other ELPs. For instance, at the ELP bulk concentration of 5.8 mg/mL, the ΔF and ΔD after rinsing were found to be approximately −27 (Hz)/~2 (×10^−6^) for ELP4, −13 (Hz)/~1 (×10^−6^) for ELP2, and then −9 (Hz)/~0.3 (×10^−6^) for ELP1. As a general comparison, Costa and colleagues [24] recently showed that deposition of their recombinant ELP, H-RGD6 (~61 kDa, 1 mg/mL in 0.15 M NaCl), onto either chitosan or gold coated QCM-D sensors resulted in large ΔF and ΔD of approximately −113 (Hz)/~18 (×10^−6^) and −67 (Hz)/~8 (×10^−6^) (7th overtone) respectively. Thus, our results appear to be in the same relative magnitudes of ΔF and ΔD as those found in the literature for similar proteins.

**Fig. 8:**
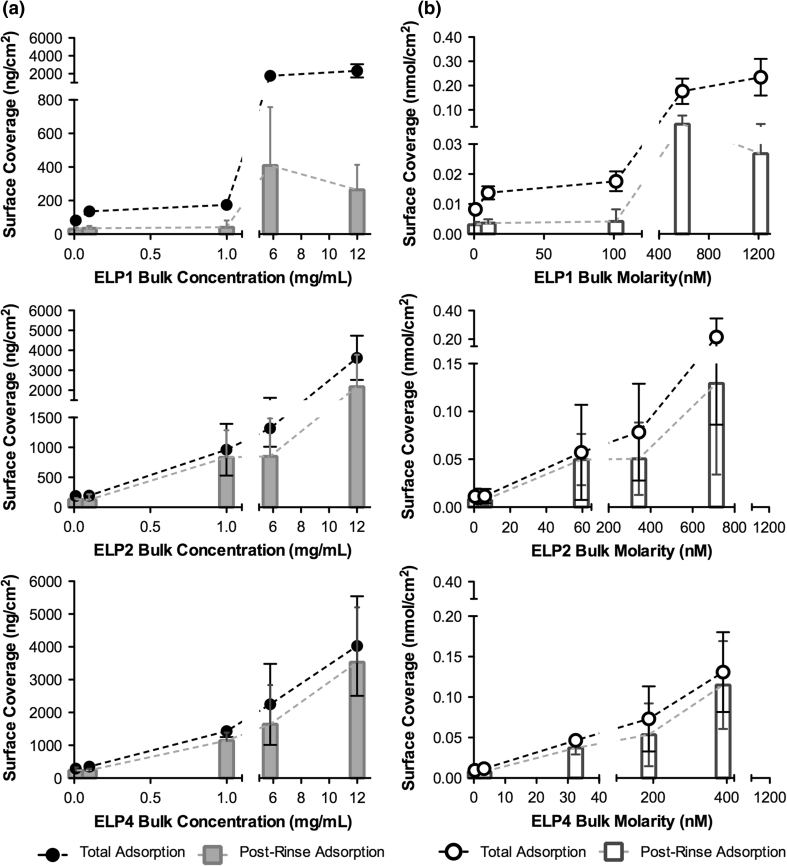
Summary of the ELP surface coverage in terms of **a** the mass adsorbed (ng/cm^2^) and **b** the moles adsorbed (nmol/cm^2^) as a function of the ELP bulk solution (mg/mL and nM, respectively) following total adsorption (*circle*) (i.e. 3 h of static adsorption) as well as following post-rinse adsorption (*bar*) (i.e. ~30 min of rinsing in PBS buffer). *Data* represented as the mean ± SD, *n* ≥ 3

From the generated ΔF and ΔD, the ELP surface coverage in terms of the adsorbed ELP mass (ng/cm^2^) and moles (nmol/cm^2^) on the surface from the range of ELP bulk solutions were estimated using either the Sauerbrey relation (when ΔD was below 5 % ΔF) or the Voigt-based model [22], as shown in Fig. 8. In particular, ΔD indicates the extent of any viscoelastic changes in the adsorbed layer during the adsorption process. If ΔD is very small (<1 × 10^−6^), this indicates that the adsorbed layer is rigid and compact. In contrast, large ΔD (>1 × 10^−6^) are commonly associated with adsorbed layers that are softer, more hydrated and contain a more flexible conformation [38, 39]. At the lower bulk concentrations, <1 mg/mL for ELP1 and <0.1 mg/mL for both ELP2 and ELP4, ΔD at the end of adsorption (i.e. static adsorption for the 3 h duration) were close to zero (see Fig. 7), suggesting the adsorbed layers were thin and rigid. Therefore, the Sauerbrey relation was used to estimate the total and post-rinse ELP surface coverage. On the other hand, at higher ELP bulk concentrations, ΔD values following adsorption were greater. This indicated that the ELP adsorbed layers were more flexible and hydrated and thus, the applicability of using the Voigt-based model [22] to estimate the ELP surface coverage. It should be noted that with both models (i.e. Sauerbrey and Voigt) it was assumed that a uniform, homogenous ELP adsorbed layer exists and that at each bulk concentration the ELP assembled as a tightly packed layer. However, it is possible that the ELPs could have adsorbed as discrete particles randomly distributed on the sensor surface generating a similar QCM-D response. Hence, the modeled values reported in this study should be viewed as approximations only.

According to the adsorption isotherms (Fig. 8) from the range of ELP bulk solutions investigated, the ELPs appear to absorb producing a minimum of a monolayer coverage following the total adsorption stage as well as in the post-rinse or the desorption stage, where any loosely bound ELP was removed. In general, each ELP isotherm shows a similar adsorption process of an initial steep rise followed by a steady increase in surface coverage. The adsorption isotherms, on a mass basis (Fig. 8a), also indicate that ELP4 adsorbs more readily to the PET-coated surface with a greater amount of adsorbed ELP4 at each ELP bulk concentration than with the other ELPs. This is particularly noticeable in the initial part of the isotherm (~1 mg/mL or lower) where the rise in the isotherm appears to be steeper for ELP4 than for the other ELPs. Interestingly, on a molar basis (Fig. 8b), the initial portions of both the ELP2 and ELP4 isotherms were comparable in terms of surface coverage resulting in a great number of adsorbed moles to the surface than for the shortest polypeptide, ELP1. Collectively, this may suggest that the longer polypeptides ELP2 and ELP4 exhibit a stronger tendency to interact with the PET-coated surface compared to the shorter ELP1, which also supports the XPS findings. In the later portion of the isotherms (Fig. 8), the variation in the reversibility of the ELP adsorbed layer (i.e. difference between the total adsorbed and post-rinse adsorbed layer) is more apparent, with lower levels observed for the longer polypeptides. In particular, at the ELP4 bulk concentration of 5.8 mg/mL (equivalent to 188 nM), ~2,248 ng/cm^2^ (or ~0.07 nmol/cm^2^) of ELP4 adsorbed to the PET-coated, while after the buffer rinse ~1,645 ng/cm^2^ (or ~0.05 nmol/cm^2^) of ELP4 remained on the PET-coated sensor. In comparison, at the ELP1 bulk concentration of 5.8 mg/mL (equivalent to 588 nM), ~1,742 ng/cm^2^ (or ~0.18 nmol/cm^2^) of ELP1 adsorbed to the PET-coated followed by ~410 ng/cm^2^ (or ~0.04 nmol/cm^2^) remaining after the buffer rinse. This finding further supports the conclusion that the stability of the ELP adsorbed layer is higher with the longer polypeptides.

**Fig. 9:**
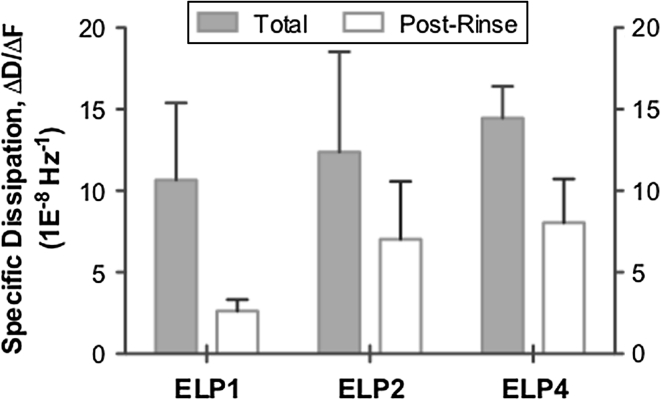
Comparison of specific dissipation (ΔD/ΔF) for the ELP adsorbed layer at the bulk concentration of 5.8 mg/mL following total and post-rinse adsorption. *Data* represented as the mean ± SD, *n* ≥ 3

To further highlight any differences in the ELP adsorbed films at the bulk concentration of 5.8 mg/mL, specific dissipation (defined as the ΔD/ΔF) was monitored to compare the relative ELP adsorbed layer viscoelastic properties after total adsorption and rinsing, as summarized in Fig. 9. This value (also referred to as normalized dissipation or aggregate viscoelasticity) is a measurement of the dissipation per adsorbed biomolecule on the surface and is commonly used in QCM-D studies as an indicator of the viscoelastic properties of the adsorbed film [40–44]. If an adsorbed film displays a lower value of specific dissipation it is attributed to a more rigid, compact film. However, a large value of specific dissipation indicates a film with a higher amount of associated water content as well as an expanded, flexible conformation [40, 42, 43]. For instance study by Dutta et al. [41] found that adsorption of poly(l-lysine) (>300 kDa, 300 μg/mL in PBS) and histone (21.5 kDa, 21.5 μg/mL in PBS) to gold coated QCM-D sensors resulted in specific dissipation values of ~8.7 × 10^−8^ and ~13.6 × 10^−8^ Hz^−1^, respectively, indicating that the poly(l-lysine) layer formed a more rigid, compact layer with less trapped water than the adsorbed histone layer. Subsequent cross-linking of the protein layers with glutaraldehyde reduced the specific dissipation values to ~1.0 × 10^−8^ and ~2.8 × 10^−8^ Hz^−1^ for the poly(l-lysine) and histone layers, respectively, demonstrating an increased layer rigidity and water content loss. Therefore, lower values of specific dissipation are likely representative of more compact and dehydrated adsorbed layers [43].

Following adsorption, comparable values of specific dissipation (between ~10–15 × 10^−8^ Hz^−1^) for each of the ELP adsorbed layers were obtained. This suggested that for the most part, each of the ELPs formed soft, hydrated adsorbed layers. However, after rinsing the longer polypeptides—ELP2 and ELP4 showed higher values of specific dissipation relative to the ELP1 adsorbed layer. For instance, the specific dissipation for the ELP adsorbed layer followed the order of ELP4 (~8.0 × 10^−8^ Hz^−1^) >ELP2 (~7.0 × 10^−8^ Hz^−1^) >ELP1 (~2.6 × 10^−8^ Hz^−1^). This finding suggests that the resultant viscoelastic properties of the ELP1 adsorbed layer differs from the other ELPs, where ELP1 is possibly adsorbing in a more rigid, dehydrated and compact conformation. Also, both ELP2 and ELP4 appear to be adsorbing in a manner that establishes softer, more hydrated adsorbed layers with presumably more flexible conformations. Furthermore, unlike the ELP1 adsorbed layer that showed approximately a fourfold decrease in the specific dissipation following the buffer rinse, the longer polypeptides displayed less than a twofold decrease, further illustrating that the longer polypeptides of ELP2 and ELP4 are capable of establishing but also potentially maintaining more hydrated, flexible adsorbed layers than ELP1.

**Fig. 10:**
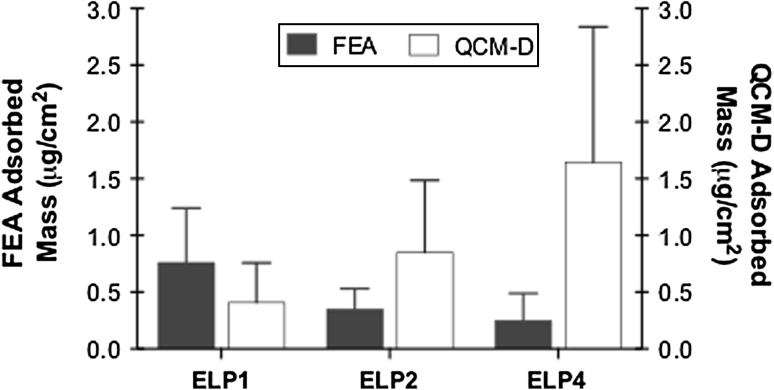
Comparison of the surface coverage (μg/cm^2^) for the three ELPs (ELP1, ELP2, and ELP4) determined using the QCM-D and a solution depletion method using an elastin specific assay (Fastin™ Elastin Assay, FEA [17])

The detected mass of the in situ QCM-D measurement is considered to be a wet mass. Consequently the measurement includes a combination of the biomolecule mass as well as the mass of the solvent that is either bound to the hydration shell (or vicinal water), and/or hydrodynamically coupled to the adsorbed film [45, 46]. Interfacial water or vicinal water is known to influence biological responses including protein adsorption [47, 48]. In general, the interfacial water surrounding a protein can be categorized as (i) buried internally (ii) ordered on the protein surface, and (iii) disordered and thereby contributing to the bulk water [49]. To estimate the contribution of the associated water content in an adsorbed layer, previous researchers have compared their QCM-D measurements to values determined from optical techniques such as surface plasmon resonance and ellipsometry [45, 46], as well as to XPS data [50] or to solution depletion measurements [51]. To obtain a general approximation of the hydration contribution to the ELP coatings, the current QCM-D surface coverage values (from an ELP bulk concentration of 5.8 mg/mL) were compared to the previously reported surface coverage values obtained under similar coating conditions but using an elastin specific assay (Fastin™ Elastin Assay, FEA) [17], as illustrated in Fig. 10. The FEA surface coverage was assumed to consider only the polypeptide content of the adsorbed film (i.e. dry mass), and as such the associated water content was approximated based on the difference between the two measurements. From this comparison, the contribution of the associated water content for the ELP adsorbed layer appeared to increase with the polypeptide length. For instance, the QCM-D adsorbed layer mass increased by a factor of 2.4 and 6.5 for ELP2 and ELP4, respectively compared to the FEA surface coverage. For the ELP1 adsorbed layer an increase in the QCM-D adsorbed mass did not occur, suggesting again that this adsorbed layer could be more compact and rigid than with the other ELPs.

At this time the source of the water content (i.e. vicinal and/or hydrodynamically coupled), observed within the ELP adsorbed films is unclear. Moreover, different trends in the hydration state of the ELPs characterized in the current study can possibly be attributed to the different surrounding states of the characterizing techniques. Nevertheless, it is evident from the current data (a combination of goniometry, XPS, AFM and QCM-D measurements) that the associated water content largely impacts the dynamic nature of the polypeptide’s adsorbed film, and subsequently as such be influencing further biological responses.

## Conclusions

In this study, we investigated the polypeptide-surface interaction of three ELPs differing in molecular weight and sequence length that were physically adsorbed to Mylar, as a means to comprehend how the surface properties of the adsorbed ELP films can influence their surface bioactivity, in particular their hemocompatibility. Adsorption of the family of ELPs increased the wettability of the hydrophobic Mylar surface, with surface wettability increasing as the ELP sequence length decreased. Chemical force microscopy analysis of the ELP-coated surfaces in PBS buffer showed no detectable hydrophobic but mostly hydrophilic interaction forces, which corresponded with the ELP surface wettability trend. ELP adsorption isotherms performed with the QCM-D indicated that the amount of adsorbed ELP increased with the polypeptide sequence length, suggesting that the longer polypeptides had a stronger tendency to interact with the hydrophobic substrate than the shorter polypeptide, ELP1. Moreover, under the coating conditions investigated, each of the polypeptides was found to adsorb to generate at minimum a monolayer coverage. The QCM-D studies also revealed that the longer polypeptides (ELP2 and ELP4) adsorbed to produce higher specific dissipation values indicating that they established films with greater structural flexibility and associated water content than the shorter polypeptide, ELP1. In addition, the stability of the coating was found to improve as the ELP sequence length increased with the greatest amount of ELP4 being retained following a shear-based desorption treatment. Clearly, varying surface properties were obtained for the three ELPs investigated, with each ELP displaying different characteristics depending on the sequence length as well as the surrounding microenvironment. Collectively, the results of this study highlight the dynamic nature of the polypeptides upon adsorption and the impact of the vicinal water layer and/or coupled water to the adsorbed film that subsequently influences the conformational state of the polypeptide film and in turn may be an important factor mediating their blood-material interaction.
